# EHMTI-0208. Positive association between -1021C/T polymorphism of dopamine-b-hydroxylase gene and level substance dependence in medication-overuse headache

**DOI:** 10.1186/1129-2377-15-S1-B33

**Published:** 2014-09-18

**Authors:** A Sergeev, J Azimova, E Klimov, K Skorobogatykh, Z Kokaeva, N Kondratieva, T Kochetkova, G Tabeeva

**Affiliations:** 1 Department of Neurology, I.M. Sechenov First Moscow State Medical University, Moscow, Russia; 2 Department of Genetics, Lomonosov Moscow State University, Moscow, Russia

## Introduction

Medication overuse headache (MOH) is a chronic headache associated with an overused of NSAIDs, combined analgesics or triptans. The pathophysiology and the risk factors of MOH are still unknown. MOH can be considered as an interaction between the chronic pain disorders and individual predisposition for the dependent behavior.

## Aims

The current study was conducted to determine the association between the dopamine β-hydroxylase (DBH) -1021C/T polymorphism and MOH.

## Methods

-1021C/T polymorphism in promoter region of DBH gene was analyzed in 44 patients with MOH with a control sample of 30 individuals without headache and without drug overuse, and with 24 patients chronic migraine without MOH and 41 episodic migraineurs. The DBH genotypes were identified by a PCR-RFLP method and clinical profile were assessed by Headache Diary and Leeds Dependence Questionnaire (LDQ)

## Results

The genotype frequencies of -1021C/T polymorphism DBH gene did not differ between the MOH and other groups. In contrast, we found that the presence of the -1021T allele was significantly associated with the monthly drug consumption and LDQ total score in the MOH group. We also found an interaction between the presence of DBH -1021T genotype and efficiency serotonin-norepinephrine reuptake inhibitors in patient with MOH.

## Conclusions

These results showed the influence of the -1021T>C polymorphism DBH gene on the number of symptomatic drug doses taken and level substance dependence (LDQ total score) in MOH, supporting a relationship between MOH and dependence-related behavior.

No conflict of interest.

